# Phytochemical Screening, Antioxidant Activity, and Acute Toxicity Evaluation of *Senna italica* Extract Used in Traditional Medicine

**DOI:** 10.1155/2023/6405415

**Published:** 2023-03-17

**Authors:** Rodrigue Towanou, Basile Konmy, Mahudro Yovo, Christian C. Dansou, Victorien Dougnon, Frédéric S. Loko, Casimir D. Akpovi, Lamine Baba-Moussa

**Affiliations:** ^1^Non-Communicable Diseases and Cancer Research Unit, Laboratory of Applied Biology Research, Polytechnic School of Abomey-Calavi, Abomey-Calavi, Benin; ^2^Research Unit in Applied Microbiology and Pharmacology of Natural Substances, Research Laboratory in Applied Biology, Polytechnic School of Abomey-Calavi, University of Abomey-Calavi, Godomey, Benin; ^3^Laboratory of Biology and Molecular Typing in Microbiology, Faculty of Science and Technology, University of Abomey-Calavi, Cotonou, Benin; ^4^Zootechnical Research and Livestock System Unit, Laboratory of Animal and Fisheries Science (LaSAH), National University of Agriculture, Ketou, Benin; ^5^Research and Training Laboratory in Applied Chemistry (LERCA), Polytechnic School of Abomey-Calavi, University of Abomey-Calavi, Cotonou, Benin

## Abstract

Medicinal plants such as *Senna italica* are increasingly used for their purgative virtues to treat stomach aches, fever, and jaundice. This study aims to screen the phytochemical compounds and to assess the antioxidant activity *in vitro* and the acute oral toxicity *in vivo* of *Senna italica* leaves. The plant was harvested, dried, pulverized, and preserved. Phytochemical screening was performed using different laboratory protocols. Ethanolic and aqueous extracts were, respectively, obtained by maceration and decoction technics. The assay for free radical scavenging was used to examine the antioxidant activity using DPPH. Acute oral toxicity was performed with aqueous and ethanolic extracts at 5000 mg/kg of body weight on female albinos Wistar rats, weighing 152.44 ± 3.68 g. Subjects were checked for any signs of mortality and macroscopy toxicity during the 14 days of the study. Biochemical and hematological parameters were measured to assess liver and kidney functions, and histological analysis of these organs was conducted. Phytochemical analysis highlighted the presence of total phenols, flavones, tannins, alkaloids, and quinone derivatives. Semiethanolic (78 *μ*g/mL), ethanolic (9.7 *μ*g/mL), and aqueous extract (9.2 *μ*g/mL) showed an interesting antioxidant activity. Biochemical and hematological parameters were normal and not significantly different (*p* > 0.05). The plant extracts did not produce any toxic effect or mortality at the provided dose. *Senna italica* extracts induced an increase in the volume of liver and kidney tissues but no necrosis. Thus, lethal dose 50 of *Senna italica* leaf extract is probably higher than 5000 mg/kg.

## 1. Introduction

According to the World Health Organization, various plant fractions and their dynamic components are used as traditional medicines by 80% of the global population [1]. One of these plants is *Senna italica*, colloquially known as agouema, an herbaceous plant or small deciduous shrub reaching 60 cm high generally with prostrate stems. Its leaves are organized in a spiral as encountered in *Leguminosae-Caesalpinioideae* family. They are glaucous, glabrous, alternate, and paripinnate with 5 to 6 pairs of obliquely oblong, obovate leaflets, and adnate at both ends with pointed apices [2]; the leaves, the ripe seeds, and the pods of *Senna italica* (*S.italica*) have always been consumed for their purgative virtues. Taken as a maceration or decoction, they may be useful to treat jaundice, stomach aches, venereal diseases, fever, and bilious crises as well as intestinal affections. The leaves are dried, pulverized, and used as a remedy for skin troubles such as ulcers and burns. The flowers are employed to purge and induce childbirth. The macerated roots are taken to treat colics and flu. The macerated roots are used to treat colic and flu. Boiled roots are used to dress wounds [3]. The infusion is smeared on eyes to relieve sores. *S. italica* roots are also a constituent of various medicines taken against indigestion, liver problems, spleen disorders, dysmenorrhea, vomiting, and nausea. In Malawi, the root infusion is used to treat infantile diarrhea. The value of *Senna italica*. as a grazing plant is not unanimous [4]. In East Africa, most domestic animals eat it, while in West Africa, they seem to avoid it. On top of the purgative effects of the mature seeds, the young seeds are consumed as an appetizer or a vegetable in the region of Sahel. In Mauritania, the seeds of *Senna italica Mill*. are smoked. Sold as “neutral henna” or “blond henna,” the leaves are also exploited as conditioner to make hair shiny. It can give hair a yellowish tint [5].

Several anthraquinones such as aloe emodin, chrysophanol, rhein, sennosides, and sennidines have been isolated from the leaves and pods of *Senna italica*. These different compounds are responsible for the purgative effect of this plant. Chrysophanol is another active component of “neutral henna.” The proportion in the leaves varies from 1.1 to 3.8% of dry weight. The pods show a much lower content than those of the leaves. In addition, the leaves contain steroids (*α*-amyrin, *β*-sitosterol, and stigmasterol) and flavonoids (kaempferol, quercetin, and apigenin) [3]. The ethanolic extract of *S. italica* plant possesses some interesting antipyretic and anti-inflammatory properties. 1,5-dihydroxy-3-methoxy-7-methylanthraquinone obtained from *Senna italica Mill*. can be used against several Gram-negative and Gram-positive bacteria. It also shows an anticarcinogenic activity *in vitro* [5]. Toxicity assays performed on rabbits and goats fed with the plant foliage were negative. Chicks and rats nourished with 10% of seed diet presented symptoms of toxicity, but no mortality sign during the 6-week trial. A 2% seed diet stimulated chick growth. Seeds produce a gum soluble in water and mainly composed of D-galactose and D-mannose [6, 7].

The ethanolic and aqueous extracts of *S. italica* leaves have free radical scavenging, antioxidant, and secondary metabolite effects and are nontoxic for rats.

To explore the phytochemical constituents of *Senna italica*, we performed phytochemical screening and determined the phenolic composition. To assess the extracts capacity for free radical scavenging, we measured their antiradical activity. To check if the extracts are toxic or not for rats, we executed the acute toxicity test on Wistar rats following the OECD protocol.

## 2. Materials and Methods

### 2.1. Medicinal Plant Extracts

An extraction of *S. italica* leaves was conducted using traditional medicine techniques, utilizing aqueous, ethanolic, and semiethanolic methods. The leaves were freshly collected from the Grand Popo (Agoué) commune in the south of Benin and identified in the Benin National Herbarium under the identification number N° YH 722/HNB. Following collection, the leaves were dried at a controlled temperature of 20 to 25°C, ground into powder, and stored in a hermetically sealed container using a proper protocol [8]. For the aqueous extract, 50 grams of *S. italica* leaf powder was boiled in 500 mL of water at 100°C. The ethanolic extracts were prepared by mixing 50 grams of leaf powder with ethanol or 50% ethanol and continuously agitating the mixture for 72 hours. The resulting macerate was filtered using the Whitman paper N°1, and the filtrates were concentrated in a rotavapor before being dried in a proofer at 50°C. The extraction yield was calculated using the following formula:

(1) $$\begin{array}{c} \%\;Yield=\frac{\left(Dry\;weight\;of\;extract\right)}{\left(Dry\;weight\;of\;extract\right)}\;x\;100. \end{array}$$

### 2.2. Phytochemical Identification

Phytochemicals present in the leaf powder were identified using a chemical method. Various tests including alkaloids, tannins, phenols, flavonoids, quinone derivatives, saponins, cyanogenic compounds, and coumarins were conducted using previously described methods [9].

### 2.3. Determination of Total Phenolic, Tannins, and Flavonoid Contents

The total phenolic compounds in the extracts obtained from 1/15 sample (w: v) of fresh nuts were measured using Folin–Ciocalteu's method, as described by singleton, six solvents (water, methanol, ethanol, water-HCl 1%, ethanol-HCl 1%, and methanol-HCl 1%) were used to determine the extraction capacity of C. nitida. The nuts were ground with a grinder, adapted to 96 well-plates, and 25 *μ*l of Folin–Ciocalteu's reagent (50% v/v) were combined with 10 *μ*l of 1 mg/ml (w/v) of the nuts extract. After incubation at room temperature for 5 min, 25 *μ*l of 20% (w/v) sodium carbonate (Na_2_CO_3_) and water were added to reach a volume of 200 *μ*l per well. Blanks were prepared using water instead of the reagent to minimize the impact of interfering compounds. The absorbance was measured at 760 nm after incubation (30 min) using a multiwell plate reader. The assays were performed in triplicate at least, and the results were expressed as microgram gallic acid equivalent per 100 grams of extract using gallic acid (0–500 *μ*g/ml) as a standard.

The total flavonoids in each sample were quantified using the aluminum trichloride method adapted to 96 well-plates [10]. Hundred microliters of methanolic AlCl3 (2%) were mixed with 100 *μ*l of appropriate dilution of the extract solution. After incubation (15 min), the absorbance was measured at 415 nm using a multiplate Epoch spectrophotometer Biotech connected to a computer with the help of “Gen5” software against a blank (mixture of 100 *μ*l methanolic extract solution and 100 *μ*l methanol) and compared to a quercetin (0–50 *μ*g/ml) calibration curve (*R*^2^ = 0.99). The flavonoid content was expressed as mg of quercetin substitutes per 100 g of extract.

The condensed tannin content was determined using the vanillin assay recommended by Belyagoubi et al. [11]. 1500 *μ*L of vanillin/methanol solution (4%, w/v) was added to 50 *μ*L of extract (S1 or S2) and followed by the addition of 750 *μ*L of 37% HCl. The sample was incubated at room temperature for 20 min, and the absorbance was measured at 550 nm against a blank. Catechin was used as a standard for calibration curve, and the total proportion of condensed tannins was calculated as mg of catechin equivalents per g of dry matter (mg CE/g DM).

### 2.4. Antioxidant Assay

The antioxidant assay of different extracts was evaluated by the 2,2-diphenyl-1-picrylhydrazyl radical (DPPH.) method, which was often used for its simplicity. It is a technic based on the reduction of an alcoholic solution of DPPH. Provided an antioxidant that gives hydrogen or a proton, the nonradical form DPPH-H was formed [12, 13]. The decoloration of DPPH radical depends on the concentration of the different extracts used. The extract-free radical scavenging activity, expressed as IC50, defines the effective concentration of the substrate that causes loss of 50% of DPPH radical activity [14, 15]. The optic densities were measured at 517 nm and used to calculate the percentage of scavenging of the DPPH radical, which was proportional to the antiradical power of the sample [16]. These IC50 are determined from curves that estimate the antioxidant activity of extract as a percentage of crude extract concentration [12]. A volume of 100 *μ*L of each extract at different concentrations was added to 1900 *μ*L of the ethanolic solution of DPPH (40 *μ*g/mL). The negative control was prepared in parallel by mixing 100 *μ*L of extraction solvent with 1900 *μ*L of DPPH solution. After incubation at room temperature and in the darkness for one hour, absorbances are taken at 517 nm using a HACH LANGE DR 3900 spectrophotometer.

The percentage of trapping was calculated by the formula:

(2) $$\begin{array}{c} Scavenging\;DPPH\;radi\;cal=\frac{{OD}_{Control}-{OD}_{Test\;sample}}{{OD}_{Control}}\times 100. \end{array}$$

### 2.5. Acute Toxicity Study

Nine male Wistar rats, aged 12 weeks and weighing an average of 152.44 ± 3.68 g, were housed in wire mesh cages measuring 50 × 30 × 20 cm^3^ upon receipt. They were kept in accordance with the experimental conditions outlined by the OECD (Organization for Economic Cooperation and Development) Guideline-423, adopted on December 23, 2001, as per previous studies [17]. The temperature of the room was maintained at 25 degrees Celsius, and artificial lighting was provided in alternating cycles of 12 hours of light and 12 hours of darkness. The rats were provided with standard granulated food from a commercial food supplier in Benin and were given access to drilling water ad libitum.

#### 2.5.1. Experimental Design

The acute toxicity study was conducted in compliance with the OECD Guidelines (Organization for Economic Cooperation and Development, Guideline-423, adopted on 17 December 2001). *Senna italica* extract was administered once to the rats as a single dose of 5000 mg per kg of body weight in accordance with the acute oral toxicity protocol of the OECD Guidelines for Chemicals Testing 423, adopted December 17, 2001, and in accordance with good laboratory practice. The rats were divided into three groups of three rats each: the first group of control rats, the second group of normal rats which received the aqueous extract of *Senna italica* via tube-feeding at a dose of 5000 mg/kg body weight, and finally, the third group of normal rats which received *Senna italica* ethanolic extract via tube-feeding at a dose of 5000 mg/kg body weight. The animals were fasted for 12 hours prior to the administration of the extracts, after which they were weighed and then the extracts were given based on their fasting body weight. The rats were observed for 14 days during the experiment to monitor their behavior. The extracts were considered toxic if 50% of the experimental rats had died.

#### 2.5.2. Hematological Analysis

Blood samples were collected in EDTA-coated tubes, and hematological parameters were determined using a Mindray hematology analyzer. The following hematological parameters were analyzed: total and differential white blood cell count (WBC), red blood cells number (RBC), red cell distribution width (RCDW), hematocrit (HCT), hemoglobin (Hb), mean corpuscular volume (MCV), mean corpuscular hemoglobin (MCH), mean corpuscular hemoglobin concentration (MCHC), platelet count (PLT), and mean platelet volume (MPV).

#### 2.5.3. Biochemical Analysis

After allowing blood samples in non-EDTA coated tubes to clot for 5 minutes, they were immediately centrifuged at 3000 rpm for 10 minutes to separate the serum for analysis. Aspartate aminotransferase (AST), alanine aminotransferase (ALT), urea, creatinine, alkaline phosphatase, gamma GT, and total cholesterol were analyzed using a chemistry analyzer (Hu-mastar 200, Germany). Electrolytes were analyzed using an electrolyte analyzer (Humate plus 5, Germany).

#### 2.5.4. Histopathological Analysis

On the 14th day of the experiment, the rats were sacrificed with thiopental (30 mg/kg) after sampling. The kidney and liver were removed, fixed in a 10% formalin solution, and paraffin-embedded. Microtome sections of approximately 3 to 5 *μ*m were made and mounted on glass slides. The sections were dewaxed in toluene and hydrated in decreasing alcohol baths. For histological analysis, the sections were stained using hematoxylin and eosin (H and E) following the standard protocol [18].

### 2.6. Ethical Considerations

The animal research guideline adopted by the ethics committee of the Research Unit in Applied Microbiology and Pharmacology of Natural Substances-University of Abomey-Calavi was followed to ensure adherence to experimental guidelines and animal welfare.

### 2.7. Statistical Analysis

The variance was analyzed using the procedure of generalized linear model with the R software version 4.2.0. The significance (*p* < 0.05) of the group factor was determined using the *F* test, and the averages were compared two-by-two using the student test.

## 3. Results

### 3.1. Phytochemical Identification

The results of the phytochemical analysis of *S. italica* leaves powder highlighted several secondary metabolites such as gallic tannins, catechic tannins, flavonols, leuco-anthocyanins, saponosides, triterpenoids, quinone derivatives, steroids, coumarins, mucilages, reducing compounds, o-heterosides, and c-heterosides. On the other hand, secondary metabolites such as anthracenes, anthocyanins, and cyanogenic compounds are absent from *Senna italica* leaves (Table 1).

### 3.2. Analysis of Flavonoid and Total Phenolic Contents

The ethanolic extract contains more total phenols (3179.91 ± 223.11) mg GAE/100 g and tannins (2.74 ± 0.15) mg·EC/g than aqueous extract polyphenol = 1497.24 ± 21.55 mg·GAE/100 g and tannins = 1.10 ± 0.04 mg·EC/g.

The flavonoid contents in aqueous extract were higher than flavonoid of ethanolic extract (Table 2).

### 3.3. Antioxidant Activity

Free radical scavenging depends on the concentrations of gallic acid, butylated hydroxytoluene, and quercetin. These curves allowed the determination of the concentrations of each synthetic compound which allows the scavenging of 50% (IC_50_) of DPPH free radicals. It turns out that the concentrations allowed to trap 50% of the DPPH radicals by the reference compounds are, respectively, 10.45 ± 1.59 *μ*g/mL; 29.98 ± 1.91 *μ*g/mL; and 71.67 ± 2.52 *μ*g/mL for quercetin, gallic acid, and butylhydroxytoluene. Then, it makes sense that quercetin had higher activity than gallic acid and BHT (Table 3).

The antioxidant activities of *S. italica* leaves were different according to extracts. The IC_50_ values of different extracts revealed medium scavenging activity from 9.33 ± 1.31 *μ*g/mL (AE) to 9.65 ± 0.28 *μ*g/mL (EE). The high values (*p* < 0.05) of IC_50_ 78.9 ± 2.29 *μ*g/mL were obtained with 50% AEE (Table 3).

### 3.4. Acute Toxicity

The mean values of several hematological parameters, namely, white blood cell (WBC) number, red blood cell (RBC) count, hemoglobin (Hb), mean corpuscular hemoglobin (MCH), mean corpuscular hemoglobin concentration (MCHC), packed cell volume (PCV) did not (*p* > 0.05) diverge significantly in all three groups. MCV of group 2 varied significantly at day 14. Similarly, the thrombocytes were significantly increased on day 14 in all three groups, especially in the two experimental groups (Table 4).

We found a significant difference (*p* ≤ 0.05) in ALT levels and a very significant difference (*p* ≤ 0.001) in alkaline phosphatase (ALP) levels. However, AST and ALT levels at the 14^th^ day for all groups were slightly lower than the ones at the beginning. At the same time, creatinine and urea levels showed no significant (*p* > 0.05) change from day 0 to day 14. However, we noted a slight increase in the creatinine concentration at day 14 in group 1 and a slight decrease in groups 2 and 3; whereas, urea levels showed a decrease at day 14 in groups 1 and 3 and an increase in group 2 (Table 5).

#### 3.4.1. Weight of Rats

**Figure 1:**
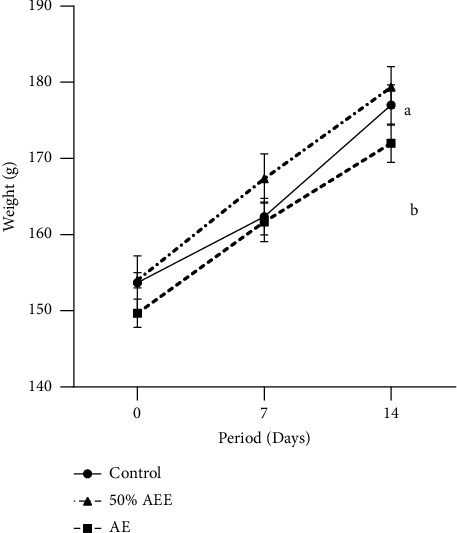
Body weight evolution during the test.

Figure 1 shows the weight variation between the experimental rats and the controls. At the beginning of the experience, the average weight in the three groups did not vary significantly. However, we found a significant increase in the average weights during the test. The rats that received aqueous extract showed the highest weight gain (198 g). The control rats showed the lowest weight gain (175 g).

### 3.5. Histological Sections

**Figure 2:**
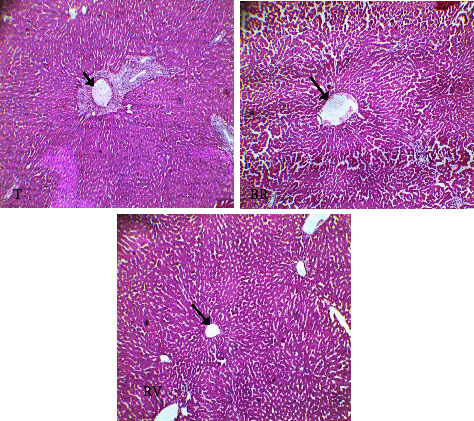
Histological sections of the liver. T: liver of the control lot. BR: liver of the lot having received the aqueous extract derived from *S. italica*. RV: liver of the lot treated with *S.italica* ethanolic extract. arrows: centro lobular vein.

**Figure 3:**
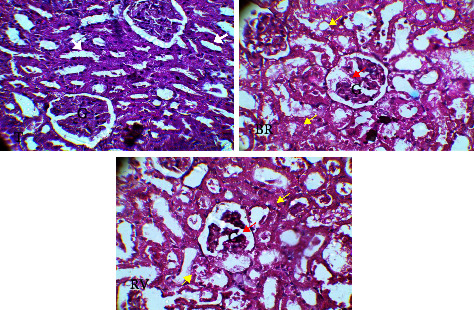
Histological sections of kidneys. T: kidney of the control lot. BR: kidney of the lot having received the aqueous extract of *S. italica*. RV: kidney of the lot having received the ethanolic extract of *S. italica*. G = Glomerulus. White arrow = renal tubules with normal morphology. Red arrow = altered glomeruli with retrieve of some podocytes and increase of Bowman's space. Yellow arrow = tubular epithelial cells detachment from the basal lamina and necrosis of several of them.

Figures 2 and 3 show the results of histological sections of the kidney and of the liver, respectively.

The diameter of the centrilobular vein was enlarged in rats having received the *S. italica* aqueous extract, whereas in rats treated with the ethanolic extract, this vein was narrowed. In all experimental rats, the volume of hepatocytes increased (Figure 2).

Analysis of the Figure 3 shows that the renal cortex was normal in all rats. The glomeruli are normal (compact) in the controls but are altered (red arrow) with the retrieval of some podocytes and increase of Bowman's space in the batches that received the extracts. The renal tubules (black arrows) in the controls had a normal morphology. Their volume had increased in the experimental rats. The tubular epithelial cells (yellow arrow) were detached from the basal lamina and several of them were necrotic (Figure 3).

## 4. Discussion

The main organ managing vital functions like digestion and detoxification of molecular compounds in the body is the liver [1]. In developing countries, the use of pharmacopeia therapies is very common because of the flora wealth. However, the chemical content and safety of certain plants used remain unknown. This work has looked for the chemical composition, the antioxidant power, and the acute oral toxicity of *S. italica* leaves extracts.

Phytochemical analysis of *S. italica* leaves powder showed steroids, alkaloids, tannins, flavonoids, saponosides, mucilages, quinone derivatives, and cardiotonic derivatives. On the other side, we did not find any traces of free anthracenes and cyanogenic derivatives. These results support the ones of [19] who also detected flavonoids, alkaloids, and steroids presence in the aqueous and methanolic extracts of *S. italica*. Tannins were not found in Dabai et al. [19], as opposed to our study. This could be due to the variations observed in the nature of the plant material, phenological state, and geographical location [19]. The present work is not fully in line with the work of Alqethami and Aldhebiani [20] who did not detect flavonoids presence in *S. italica* fruit, but did find tannins and saponins as in our study [20].

The nonappearance of certain secondary metabolites in our results could be justified by the seasonal variations. They can affect chemical composition but also biological activity of plants [21]. The results of this work agree with those of [22] who did not detect the presence of cyanogenic derivatives in the leaves of *S. italica*.

Phenolic substances are recognized for their numerous health benefits. Various pharmacological activities (antioxidant, anticancer, and antimicrobial activities) are attributed to them [23]. The study showed that the aqueous extract (*p* < 0.001) has the highest proportion of total phenol (1497.24% ± 21.55) and of flavonoids (1278.11% ± 21.23) (*p* < 0.0001). Besides, the highest percentage of condensed tannin (2.74 ± 0.15) was attributed to the ethanolic extract (Table 2). Our results are similar to those obtained by Dah-Nouvlessounon et al. [10]. They obtained the highest content of total flavonoids (561.69 ± 22.10 *μ*gQE/100 g) in *G. kola*. These findings prove that total flavonoids represent an important part of the total phenolic composition of *Senna italica*. Previous studies have reported many herbs as an excellent source of phenolic molecules recognized for their antioxidant effects [24, 25].

The present work shows a progressive rise in the trapped DPPH percentage. It goes up to 80%. The aqueous extract of this plant was the most active with an IC50 equal to 10 *μ*g/mL and ensued by the semiethanolic extract (66 *μ*g/mL) and by the ethanolic extract with an IC50 value equal to 78 *μ*g/mL. The concentrations in semiethanolic and ethanolic extracts of *S. italica* leaves diverged from 9.2 *μ*g/mL to 78 *μ*g/mL. This is not in line with the results of [26] which pointed the ethanolic extract as the best source of antioxidants. The highest IC50 value was given by the semiethanolic extract. This proves the low antiradical activity of semiethanolic extract compared to others. The aqueous extract showed the highest activity with an IC50 value = 9.2 *μ*g/mL tailed by the ethanolic extract. The ethanolic and aqueous extracts of *S. italica* leaves showed higher antiradical activities than the controls used in this work.

Flavonoids, which pertain to phenolic compounds, are considered as potential antioxidant sources. They have the ability to reduce free radical species and reactive forms of oxygen. The reducing power of free radicals is explained by the shield effect of flavonoids. The low redox potential that makes flavonoids thermodynamic has also been attributed to their shielding effect. The transfer of the hydrogen atom generated by the antioxidant reaction gives rise to a peroxyl radical. According to the work of Mokgotho et al. [27], the antioxidant power of *S. italica* is linked to its content in resveratrol. For other authors, the use of *S. italica* in traditional care was due to its antibacterial, antioxidant, antidiabetic, and hypertensive properties [28–31].

For the acute oral toxicity of *S. italica* leaves extracts, after gavage of the rats with the ethanolic and aqueous extracts of *Senna italica*, we found neither mortality nor morbidity signs. Not a single moribund animal was obtained throughout the 14 days of experiment. This supports the results of [6]. We did not observe any change in the functioning of the skin, eye, hair, and respiratory system. Behavioral and physical signs of toxicity such as sleep disturbance, seizure, breathing, restlessness, or hyperactivity were also absent. These results give evidence for the nontoxicity of the ethanolic and aqueous extracts of *S. italica* after administration at 5000 mg/kg of body weight.

The oral ingestion of ethanolic and aqueous extracts of *S. italica* at 5000 mg/kg did not affect the normal growth of the experimental rats as shown by the evolution of the weight gain (Figure 1). However, a gradual change in their weight was underlined. According to mean weight values analysis, the weights of the rats of the three groups at day 0 and day 14 did not differ significantly. Considering the extract impact on weight gain, our results are similar to the ones of Frimpong and Nlooto [29].

To assess the extracts effects on the function of the rats's vital organs, certain hematological parameters were measured. The white blood cells (WBCs) number, hemoglobin (Hb), red blood cells (RBCs), hematocrit (Hte), the mean corpuscular volume (MCV), mean corpuscular hemoglobin concentration (MCHC), mean corpuscular hemoglobin content (HCM), platelets (PLTs) were checked for the rats. Table 3 sums up the results obtained about the hematological parameters. We found that the count of white blood cells (WBCs), hemoglobin (Hb), red blood cells (RBCs), hematocrit (Hte), and mean corpuscular hemoglobin concentration (MCHC) did not increase significantly (*p* > 0.05) in all three groups as opposed to mean corpuscular volume (MCV) which increased on day 14 compared to day 0. There is a decline in the count of red blood cells (RBCs), an anemia and an increase in case of exaggerated production or loss of liquid [32]. Platelet count (PLT) increased significantly on day 14 compared to day 1 in group 1 (*p* ≤ 0.05). It also increased very significantly (*p* ≤ 0.0001) in the two other groups on day 14. The platelet count allows the detection of a bleeding, infectious, or inflammatory risk after huge bleeding [32]. The thrombocytes count variations observed in our case would be linked to physiological growth of the rats. Statistical analysis showed no significant difference (*p* > 0.05) between the hematological parameters of experimental rats having received the aqueous extract or the ethanolic extract and control rats for the other parameters. There was also no significant variation in these parameters in each group between the first day and the fourteenth, despite the first observations. The high values of MCV, CHM, and MCHC indicate the presence of macrocytic normochromic red blood cells, while a decrease points to the presence of hypochromic microcytic RBC [30]. Apart from the significant variation in MCVs observed in batch 2 on day 14, in our case, there was no other significant behavior. This shows that the RBC of the rats were normocytic-normochromic and, therefore, that the ethanolic extract had no noxious effect on the RBC of the rats at 5000 mg/kg of body weight. In conclusion, the ethanolic and aqueous extracts of *S. italica* had no toxic effect on blood platelets and the aqueous extract of *S. italica* had no toxic effect on MCV. However, various factors related to the subject and its environment could be responsible for the nonsignificant variability recorded in this study and the observations related to the cell variability, in particular lifespan [33].

Several biochemical markers, namely, glucose, transaminases (AST and ALT), urea, creatinine, alkaline phosphatase (ALP), and gamma GT were also measured to assess the impact of ethanolic and aqueous extracts of *S. italica* on rats' vital organs. Table 5 shows the average concentrations of these parameters in the experimental group with the time. We obtained no significant difference (*p* > 0.05) between the levels of these markers in the groups on day 0 and 14, except on day 14 for group 2 (Table 5). There was a substantial variation (*p* ≤ 0.05) in ALT level and a highly significant difference (*p* ≤ 0.001) in alkaline phosphatase (ALP). However, AST and ALT levels on day 14 for all groups of rats were slightly lower than on day 0, as were creatinine and urea levels. The findings revealed no significant change (*p* > 0.05) between day 0 and 14. However, we noted a slight increase by day 14 in the creatinine level in group 1 and a slight decrease in groups 2 and 3, while urea levels decreased in groups 1 and 3 and increased in group 2. Creatinine and urea levels are good indicators of kidney function [34].

The data collected about biochemical parameters revealed no significant variation between the levels of AST, blood glucose, urea, gamma GT, and creatinine for the experimental rats exposed to the extracts compared to controls. Among the biochemical markers covered, transaminases (AST and ALT) are normally found in many cells' cytoplasm and mitochondria, mainly in the liver, heart muscle, and skeletal muscle. However, their concentrations are lower in the pancreas, kidney, and erythrocytes [35]. Therefore, increased serum AST and ALT levels indicate liver toxicity. This occur generally in the blood when the permeability of liver cells is impaired or when necrosis take place.

AST and ALT are hepatic enzymes insuring the chemical transfer of an amine group to other molecules in the liver [34]. The activity of these enzymes is relative to the degree of damage [35]. Therefore, they are two relevant indicators of hepatotoxicity [36, 37].

An increase in two-or three-folds range of ALT levels implies hepatic cytolysis [32]. An increase in serum AST activity indicates a traumatic, an inflammatory or degeneracy due to plasma membrane damage and cellular necrosis [37, 38]. The significant change in ALT levels seen in group 2 highlights a hepatic cytolysis. Inflammation and tissue degeneration noticed in rats treated with *S. italica* aqueous extract at 5000 mg/kg of body weight did not affect hepatocytes. Therefore, we can deduce that the *S. italica* ethanolic extract did not cause any damage on the liver of rats at 5000 mg per kg of body weight.

Renal function was assessed through serum ureal and creatinine concentrations. Creatinine and urea are important markers of the kidney function [39, 40]. These metabolism products have a constant level under normal conditions [41]. Renal impairment is reflected by their decrease or increase [42]. Pritchard and his colleagues had shown that a reduction in serum creatinine could indicate cachexia. Looking at the serum urea concentration, its rise can sign a nephropathy, dehydration, electrolyte imbalance, hypo-albuminemia, and tissue catabolism [42]. Because these parameters did not vary significantly in experimental rats as opposed to controls, we came to the conclusion of a normal kidney function. In sum, since no significant variation in AST, gamma GT, blood glucose, urea, and creatinine levels was noted, we can deduce that the ethanolic extract of *S. italica* is not toxic to the liver and kidneys in rats at 5000 mg/kg of body weight. *S. italica* ethanolic extract administered to rats in a proportion of 5000 mg/kg of body weight have not caused concomitant changes in the number of white blood cells and red blood cells. The first are essential to fight infection and develop resistance to infection after a prior exposure or vaccination. They consist of monocytes, lymphocytes, and granulocytes [43]. In the case of infection, inflammation, cancer or leukemia, the leucocytes count is increased and can be reduced by the bone marrow failure, liver disease, splenomegaly, autoimmune diseases, and certain drugs. Their levels are not very different in treated rats in comparison to the control group. This demonstrates that the *S. italica* ethanolic extract presents no toxicity for the white blood cells of rats at 5000 mg/kg of body weight.

*S. italica* aqueous extract administered at 5000 mg/kg body weight produced significant change in ALT levels indicating liver cytolysis, inflammation, tissue degeneration, and thrombocytosis in administered rats. This explains the switch of the hemostasis system. Moreover, there was a significant variation in the alkaline phosphatase level of the rats having received 5000 mg/kg of body weight of *S. italica* aqueous extract. We can then summarize that the ethanolic extract was not toxic at a limit dose of 5000 mg/kg of body weight, while the aqueous extract of *S. italica* would be toxic only at a dose lower than 5000 mg/kg of body weight.

## 5. Conclusions

The *Senna italica* leaves contain various pharmacologically active compounds including phenolics, derivated quinone, and flavonoids, which are good antioxidant, anti-inflammatory, and protective. *S. italica* leaves are nontoxic at the dose of 5000 mg/kg of body weight. Thus, the leaves of *S. italica* have a powerful antioxidant activity. They can be used orally as traditional medicine. *S. italica* leaves constituted then a useful phytobiotic resource which can promote human health. Dose studies are, however, necessary depending on the type of pathology.

## Figures and Tables

**Table 1:** Phytochemical screening of *S. italica* leaves powder.

Secondaries metabolites	*Senna italica*
Catechic tannins	+
Gallic tannins	+
Flavonoids	+
Anthocyanins	−
Leuco-anthocyanins	+
Quinones derivatives	+
Saponosides	+
Triterpenoids	+
Steroids	+
Mucilages	+
Cyanogenic compounds	−
Coumarins	+
Reducing compounds	+
Free anthracenics	−
O-heterosides	+
C-heterosides	+
Cardiotonic derivatives	+
Alkaloids	+
Sterols and triterpenes	−

(+) = presence, (−) = absence.

**Table 2:** Total phenolic, flavonoid, and condensed tannin contents of *S. italica* leaves.

Plant species	Parameters		
	Total phenolic (mg GAE/100 g)	Total flavonoids (mg QE/100 g)	Condensed tannins (mg CE/g)
Ethanolic extract	3179.91 ± 223.11	898.36 ± 4.04	2.74 ± 0.15
Aqueous extract	1497.24 ± 21.55	1278.11 ± 21.23	1.10 ± 0.04
*p* value	<0.001	<0.001	<0.001

**Table 3:** Antioxidant activity of *S. italica* leaves extracts.

	*Senna italica* leaves extracts			Control		
	AE	50% AEE	EE	Quercetin	Gallic acid	BHT
DPPH radical scavenging (IC_50_ value, *μ*g/mL)	9.33 ± 1.31	78.9 ± 2.29	9.65 ± 0.28	10.45 ± 1.59	29.98 ± 1.91	71.67 ± 2.52

DPPH = 2, 2′-diphenyl-1-picrylhydrazyl; AE = aqueous extract; 50% AEE = 50% aqueous ethanolic extract; EE = etha-nolic extract; BHT = butylated hydroxytoluene; IC_50_ = half-maximal inhibitory concentration.

**Table 4:** Hematological parameters of rats.

Indexes	Days	Control	AE	EE
WBC (×10^9^/L)	D0	13.96 ± 0.73/b	9.03 ± 1.88/b	10.46 ± 0.43/c
	D14	14.20 ± 0.00	11.90 ± 2.07	14.90 ± 0.75^*∗∗*^/

RBC (×10^6^/mm^3^)	D0	6.17 ± 0.40	6.88 ± 0.16	7.25 ± 0.31
	D14	6.65 ± 0.14	6.24 ± 0.02	7.58 ± 0.11

Hb (g/dL)	D0	13.43 ± 0.48	13.10 ± 0.75	13.00 ± 0.60A
	D14	13.35 ± 0.26	13.10 ± 0.05	14.65 ± 0.08B

PCV (%)	D0	41.42 ± 0.89/b	43.43 ± 0.69/a	41.60 ± 1.91/b
	D14	43.25 ± 0.20^*∗*^/a	46.35 ± 0.26^*∗*^/b	50.25 ± 0.95^*∗∗∗*^/c

MCV (fL)	D0	62.2 ± 1.12	60.76 ± 0.80A/a	56.33 ± 0.76/b
	D14	61.91 ± 1.14/a	74.25 ± 0.72B/b	66.35 ± 2.22^*∗∗∗*^/a

MCH (pg)	D0	19.73 ± 0.82	19.96 ± 0.35	18.40 ± 0.30
	D14	19.80 ± 0.11/b	21.00 ± 0.00^*∗*^/a	19.35 ± 0.37/b

MCHC (g/dL)	D0	31.96 ± 0.96	33.00 ± 0.60^*∗*^/	32.06 ± 0.43
	D14	32.00 ± 0.11/a	28.30 ± 0.28/b	29.15 ± 0.37/b

PLT (×10^3^/mm^3^)	D0	775 ± 39.06/a	902.33 ± 16.69^*∗∗∗*^/b	751.66 ± 11.02^*∗∗*^/a
	D14	734 ± 3.46/a	656.00 ± 32.90/b	528.00 ± 21.32/c

AE: aqueous extract group; EE: ethanolic extract group; RBC: red blood cells, MCV: mean corpuscular volume, PCV: packed cell volume; MCHC: mean corpuscular hemoglobin concentration; MCH: mean corpuscular hemoglobin; WBC: white blood cell; PLT: thrombocytes. Means with letters a; b; c in the same row are different (*p* < 0.05); Means with ^*∗*^; ^*∗∗*^; ^*∗∗∗*^ in the same column are different for D0 and D14 of the same parameter. ^*∗*^=*p* < 0.05; ^*∗∗*^=*p* < 0.01; ^*∗∗∗*^=*p* < 0.001.

**Table 5:** Biochemical parameters of rats.

Indexes		Control	AE	EE
Glucose (g/L)	D0	0.64 ± 0.02	0.55 ± 0.03	0.60 ± 0.01
	D14	0.79 ± 0.06/a	0.66 ± 0.02b	0.69 ± 0.01/b

Urea (g/L)	D0	0.21 ± 0.02	0.24 ± 0.03	0.25 ± 0.02
	D14	0.20 ± 0.00/a	0.44 ± 0.02^*∗∗*^/b	0.22 ± 0.02/a

Creatinine (mg/L)	D0	10.33 ± 0.84	10.07 ± 0.26	9.78 ± 8.86
	D14	11.67 ± 0.33	9.05 ± 0.54	8.78 ± 0.69

ALT (UI/L)	D0	56.96 ± 5.05^*∗∗∗*^/a	60.06 ± 4.16^*∗∗∗*^/b	36.68 ± 1.92^*∗∗*^/c
	D14	14.66 ± 0.23/a	15.49 ± 1.74/a	24.73 ± 3.04/b

AST (UI/L)	D0	21.56 ± 0.55^*∗*^/a	25.66 ± 1.18/b	31.01 ± 3.45^*∗∗*^/c
	D14	18.43 ± 0.06/a	24.32 ± 2.15/b	27.40 ± 1.11/c

ALP (UI/L)	D0	460.12 ± 21.18/a	495.69 ± 60.21/b	503.37 ± 28.80^*∗*^/c
	D14	438.65 ± 3.43/a	895.68 ± 6.21^*∗∗∗*^/b	577.46 ± 107.29/c

ƴGT(UI/L)	D0	3.61 ± 0.25/a	4.66 ± 0.39^*∗*^/b	4.82 ± 0.47/b
	D14	3.93 ± 0.05/a	3.30 ± 0.04/a	4.50 ± 0.39/b

AE: aqueous extract group; EE: ethanolic extract group; AST: aspartate aminotransferase; ALT: alanine aminotransferase; ALP: alkaline phosphatase; *ƴ*GT: gamma glutamyl transferase. Means with letters a; b; c are different (*p* < 0.05); means with ^*∗*^; ^*∗∗*^; ^*∗∗∗*^ are different for D0 and D14 of the same parameter. ^*∗*^=*p* < 0.05; ^*∗∗*^=*p* < 0.01; ^*∗∗∗*^=*p* < 0.001.

## Data Availability

No additional information is available for this paper.

## Supplementary Materials

Supplementary Materials EQUATOR network study checklist.
