# Supplementary material for: Phytochemical Screening, Antioxidant Activity, and Acute Toxicity Evaluation of Senna italica Extract Used in Traditional Medicine
Source: J Toxicol. 2023 Mar 17;2023:6405415. doi: 10.1155/2023/6405415 (PMC10038741; doi:10.1155/2023/6405415)
Supplement: Supplementary Materials — EQUATOR network study checklist. [file 6405415.f1.docx]

| EQUATOR network study checklist | |
| --- | --- |
| Study design 1 | Phytochemical screening  Anti-radical activity  Acute oral toxicity  a) for the toxicity 3 groups were constituted; 1 control group and 2 experimental groups  b) each group is composed of 3 female rats |
| Sample size 2 | a) there are 3 rats per group and 3 groups so 9 rats were used in total during the acute oral toxicity.  b) the sample size was chosen according to the OECD protocol, guideline N°423 |
| Inclusion and  Exclusion criteria 3 | a) The criteria used to include and exclude animals are those described in the OECD protocol No. 423, of which the rat must be a female rat weighing 180g ± 20  b) male rats and female rats weighing more than 200g and less than 160g are systematically excluded  c) for each group, the weights of the rats were taken and some blood tests were also carried out. |
| Randomisation 4 | Experimental units were randomized to obtain a complete randomized block.  Animals belonging to the same experimental unit were marked on the tail with permanent markers for identification. |
| Blinding 5 | The head of the non-communicable diseases and cancer unit was aware of the allocation of the groups at the different stages of the experiment (during the allocation, the conduct of the experiment, the evaluation of the results and the analysis of the data). |
| Outcome measures 6 | weight of the rats, blood count, urea, creatinemia, blood sugar, alkaline phosphatase transaminases and gama GT. |
| Statistical methods 7 | A one-factor Analysis of Variance was performed with R software to compare the different groups at each level.  The Turkey test was then performed in case of significant difference (p<0.05).  The homogeneity of the variance and the normality were previously verified by the chapiro wilk and leven tests. |
| Experimental animals 8 | a) Albino rats of Wistar source (Rattus norvegicus) weighing 180 ± 20 g and aged 12 weeks of female sex were used  b) They were obtained from the animal house of the Institute of Biological and Applied Sciences (ISBA) of the Faculty of Health Sciences (FSS) of Cotonou (Benin). |
| Experimental  Procedures 9 | 3 groups of rats were formed with 3 rats per group. All rats were weighed at D0, D7 and D14. Rats were fed with a standard pellet diet provided by Véto Service SA (GVS) in Benin and fed ad'libitum with water.  Hematological analysis was performed on blood samples collected in tubes containing the anticoagulant EDTA using a Mindray hematology machine.  Blood samples collected in tubes without anticoagulant were centrifuged at 3000 rpm for 10 min. The collected sera, stored at -20°C, were used for the determination of biochemical parameters.  Representative sections of liver and kidney were made and fixed in histological cassettes and processed according to the histological technique (circulation) for twelve hours. |
